# Propranolol Hydrochloride Buccoadhesive Tablet: Development and *In-vitro* Evaluation

**DOI:** 10.22037/ijpr.2019.13866.13346

**Published:** 2020

**Authors:** Seyedeh Maryam Mortazavi, Seyed Alireza Mortazavi

**Affiliations:** *Department of Pharmaceutics,* *School of Pharmacy, Shahid Beheshti University of Medical Sciences, Tehran, Iran.*

**Keywords:** Propranolol HCl, Buccoadhesive tablet, Mucoadhesive polymers, Mucoadhesive strength, Duration of mucoadhesion

## Abstract

Propranolol HCl is a beta blocker commonly used worldwide; however, it shows a low bioavailability due to its extensive first-pass metabolism. To overcome this problem, a novel drug delivery system such as buccoadhesive system might be helpful. The aim of the present investigation is to prepare the buccoadhesive tablet of propranolol HCl using different mucoadhesive polymers. Buccoadhesive tablets containing drug, lactose, and polymers such as HPMC K4M, carbomer 934P, PEO 8000000 and PEG 6000, in various concentrations, were prepared. The tablets were evaluated in terms of weight variation, thickness, hardness, friability, and mucoadhesive strength. Among thirteen prepared formulations, seven of them which had better physicochemical properties and mucoadhesive strength were undergone the release and swelling tests. Finally, two formulations were selected and uniformity, drug content, duration of mucoadhesion, and kinetic studies were performed for them. All polymers except PEG 6000 were appropriate for being used in buccal mucoadhesive systems. Formulation F_1_ was considered as the most desirable formulation as it exhibited appropriate mucoadhesive strength (43.93 ± 12.4 g), extended duration of mucoadhesion (19.15 ± 0.29 h) and suitable swelling ability while having a prolonged drug release over 12 h. Although the efficiency and mucosal irritation of propranolol HCl buccoadhesive tablets should be monitored under the *in-vivo* conditions, however, based on the results, it seems that such tablets can be considered as an alternative route to bypass the first pass metabolism of propranolol HCl.

## Introduction

The oral route is generally preferred to the other routes for drug administration (1). But besides the advantages, there are also some disadvantages, including presystemic clearance in liver and instability in the acidic environment (2). These problems caused to develop alternative administration routes such as mucosal routes; using buccal mucosa for drug delivery is one of them (3). Having a lot of blood vessels and decent permeation leads to attention to buccal mucosa as a route for drug administration (4). In addition, it is possible to terminate the delivery of drug molecules in the case of toxicity, due to the buccal cavity is easily accessible (5). 

Buccoadhesive systems are one of the various systems that have been developed to be used in the buccal cavity. The main benefit of these systems is increasing the time of residence at the site of absorption, consequently reducing the number of applications as well as increasing patient compliance (6). From the technical point of view, mucoadhesive drug delivery systems are able to control the release of drug molecules over the time (7). Furthermore, there is a possibility of local and systemic drug delivery using buccocoadhesive systems. Additional advantages of the buccoadhesive systems include bypassing the first-pass effect and ease of use in non-conscious patients (8). Of course, this route has some drawbacks too, such as small surface area, low permeability compared to the sublingual membrane, and diluting the drug by high salivation rate. However, the advantages of this administration route are more than its disadvantages (3).

 Bioadhesion is known as the interfacial phenomenon in which some special polymers are attached to the biological surface. The mucoadhesion also means attachment of polymers to mucous membrane coated with a thin layer of mucus (9, 10).

Propranolol HCl, a non-selective beta blocker, is widely applied in the treatment of hypertension, angina pectoris, arrhythmia, thyrotoxicosis, and migraine prophylaxis. However, due to extensive first-pass effect, propranolol HCl has low systemic bioavailability. The half-life of this medicine is approximately 3 to 5 h and it has low molecular weight (295.81 g/mol), therefore propranolol HCl is a decent candidate as a drug model for buccoadhesive drug delivery system (11).

In order to prepare mucoadhesive tablets, various mucoadhesive polymers can be employed, for example mucoadhesive buccal tablets based on chitosan/gelatin microparticles for delivery of propranolol HCl were successfully prepared by Abruzzo *et al*. (12). 

In this study, buccoadhesive tablets have been developed using HPMC K4M, carbomer 934P, polyethylene oxide 8000000 (PEO), and polyethylene glycol 6000 (PEG). Due to lack of this dosage form in the national and international drug market, the aim of this study was to prepare buccal mucoadhesive tablets capable of remaining in contact with the adhesion site for a reasonable time as well as producing optimum drug content release.

## Experimental

*Materials*

Propranolol HCl was received as gift sample from Darou Pakhsh Pharmaceutical Co., Iran. Hydroxypropyl methylcellulose K4M (HPMC K4M) and polyethylene glycol 6000 (PEG) were provided by Colorcon Pharmaceutical Co., England. Carbomer 934P was supplied by ICN., Germany. Polyethylene oxide 8000000 (PEO) was from Sigma Aldrich., USA. Lactose was obtained from BF Goodrich Co., Germany. Potassium dihydrogen phosphate, perchloric acid 70%, and sodium hydroxide were purchased from Merck., Germany. Ethanol and methanol were purchased from Bidestan Chemical Co., Iran.

*Preparation and characterization of propranolol HCl buccoadhesive tablets*

Initially, the flow and compressibility of propranolol powder were evaluated and revealed propranolol hydrochloride had suitable flow and compressibility. The tablets were then prepared by direct compression method. The weight of each tablet was 160 mg. The various polymers such as HPMC K4M, carbomer 934P, polyethylene oxide 8000000 (PEO) and polyethylene glycol 6000 (PEG), in various concentrations, were applied. Lactose was applied as filler in all formulations. The drug, polymer, and filler were physically blended and then compressed by the flat-faced punch of 9 mm diameter. Initially, 10 formulations were prepared, then considering the higher mucoadhesive strength of HPMC K4M and carbomer 934P, the formulations based on physical mixture of these two polymers were also prepared. The quality control tests including weight variation, thickness, hardness, friability, and mucoadhesive strength test were performed for the whole 13 formulations. The composition of polymers and lactose within each formulation is given in Table 1.

In the next stage, formulations which had better physicochemical properties and mucoadhesive strength were chosen. They included; F_1_ and F_2_ (containing 40% and 30% HPMC K4M respectively), F_4_ and F_5_ (containing 40% and 30% carbomer 934P respectively), F_7_ (containing 40% PEO 8000000), F_11_ (containing 12% HPMC K4M and 28% carbomer 934P), and F_13_ (containing 28% HPMC K4M and 12% carbomer 934P). These seven formulations were subjected to further examinations including determination of *in-vitro* drug release properties and swelling index. Finally, two formulations among those seven formulations were selected and uniformity of dosage unit, drug content, duration of mucoadhesion, and kinetic studies were performed for them.

*Measurement of the mucoadhesive strength*

Fresh sheep buccal mucosa was obtained from a local slaughterhouse. Tissue was gently rinsed in water and after removing the connective and adipose tissue, was cut into small pieces. Tissue pieces were carefully placed on pieces of nylon and put into the freezer. The consumption of fresh tissue should be avoided because this tissue has not yet lost its contractions and is not able to create a smooth surface. Therefore, after a minimum of 1 day, tissue was removed from the freezer and brought to ambient temperature. After this period, the mucosal tissue of the sheep was placed within the phosphate buffer for an hour, so that perfectly hydrated and be ready for testing the mucoadhesive strength.

**Figure 1:**
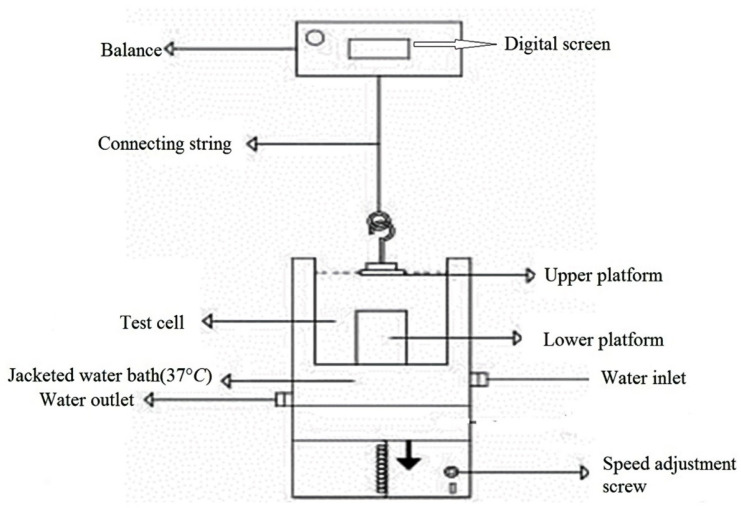
A schematic drawing of the apparatus used for measuring the *in-vitro* mucoadhesive strength of propranolol HCl formulations

To assess the mucoadhesive strength of prepared propranolol HCl tablets, we used an in-house apparatus (13, 14). The schematic drawing of this apparatus is illustrated in Figure 1.

Using a small amount of cyanoacrylate glue, the mucoadhesive tablet was bonded to the surface of the upper platform. It should be noted that only tablet contact surface with the upper platform should be smeared with glue and the other tablet surfaces are free of glue. The mucosal tissue was then placed on the surface of the lower platform (so that the surface of the mucous tissue is upwards). Phosphate buffer (pH 6.8) was poured into the test cell, so that covered the surface of the buccal mucosa. Using a water bath, the temperature of contents in the test cell was kept at 37 °C in total procedure time. After that, the tablet was placed on the mucosal surface and mild power by the fingertips was applied to it for 1 min symmetrically. The lower platform was then gradually moved down at a speed of 2 mm/min, this practice continued until complete separation of the tablet from mucosal tissue. The maximum force needed to separate the two platforms from each other was considered as the mucoadhesive strength of the tablet. Each experiment was run in triplicate, and the results were expressed as mean ± SD.

*Determination of in-vitro drug release profiles*

Initially, considering that the UV spectrophotometry method was used to determine the amount of the released drug of tablets during the release test, and that the propranolol HCl UV absorbance may be affected by added excipients in the formulation, excipient effect on propranolol HCl UV absorbance was studied. First, UV spectrum of propranolol HCl was evaluated individually. Lactose, as filler, was used with the maximum amount of 30% of the total weight of the tablet in the formulations. If this entire amount dissolves in dissolution medium, creates concentration equal to 0.053 mg/mL. This concentration was prepared in phosphate buffer and the UV-spectrum was plotted in the range 200-400 nm. This action was also performed for other excipients.

The *in-vitro* drug release studies were performed by USP dissolution apparatus 1 (rotating basket). The speed of the apparatus was considered 50 rpm. The vessels of the mentioned apparatus were filled with 900 mL phosphate buffer (pH 6.8) and the temperature was kept at 37 ± 1 °C. Five milliliters aliquots of the release medium were withdrawn at 15, 30, 45, 60, 90, and 120 min and then at every 1 h to 12 h and they replaced each time by the same volume of fresh phosphate buffer. The test was repeated 3 times for each sample and the absorption of the drug in each sample was measured with ultraviolet-visible spectrophotometry at a λmax of 291 nm. In order to convert absorbance to the amount, a linear calibration curve was used. Certain concentrations of 0.01, 0.03, 0.05, 0.07, 0.09, and 0.11 mg/mL of propranolol HCl in phosphate buffer were prepared. Finally, the equation ABS = 17.344C + 0.0418 was applied.

*In-vitro swelling study*

The test was conducted to determine the amount of water absorption and swelling of the polymer which affect drug release. Buccal tablets were weighed individually (W_1_) and placed separately in phosphate buffer (50 mL, pH 6.8, 37 ± 1 °C). At predetermined time intervals (0.5, 1, 2, 3, 4, 5, 6, 7, 8, 9, 10 h), the tablets were removed from the buffer, reweighed (W_2_), and the swelling index (SI) was calculated using the Equation 1:

$\mathrm{SI}=\frac{\mathrm{W2}-\mathrm{W1}}{\mathrm{W1}}\times 100$

 (Equation 1)

All measurements were performed in triplicate and average values ± SD were reported.

*Determination of drug content*

Since there is no pharmacopeia monograph for mucoadhesive propranolol HCl tablet, the BP monograph for propranolol HCl tablet was applied to determine drug content. A total of 20 tablets were randomly selected and powdered completely. Then a portion of powder equivalent to 20 mg of active ingredient was removed and dispersed in 20 mL of water. Fifty milliliter of methanol was added and the combination was then stirred for 1 h (for ordinary tablets, due to lack of mucoadhesive polymer, this time is 10 min). In the next step, methanol was added to bring the volume up to the final 100 mL. Following, the dispersion was passed through a glass filter. Ten milliliter of filtrate was removed and diluted with methanol to 50 mL. The UV absorbance of the resulting solution was measured.

*Determination of uniformity of dosage unit (weight variation)*

The purpose of this test is to ensure the consistency of dosage units. According to the United States Pharmacopeia, this test is performed when the active ingredient is more than or equal to 25 mg and more than or equal to 25% of tablet weight. Initially, ten tablets from each formulation were weighed individually and then the average weight was calculated. Following that, drug content of individual tablet was calculated and finally, the Acceptance Value (AV) was calculated (15).

*Assessment of duration of mucoadhesion*

**Figure 2:**
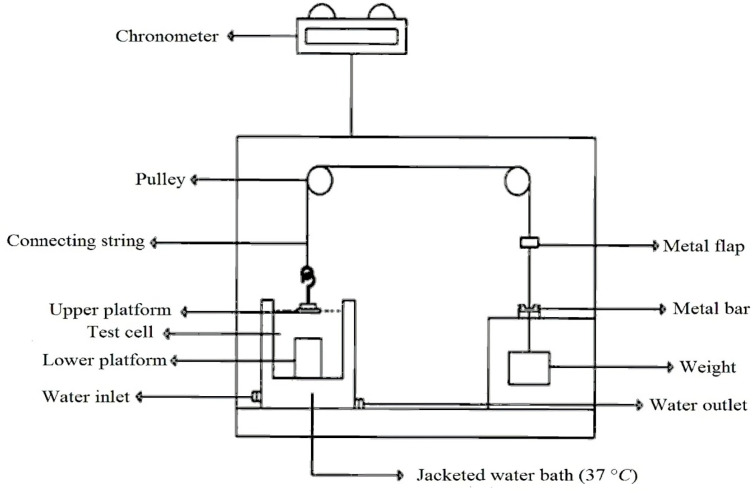
Schematic drawing of one compartment of the apparatus used for assessing the duration of mucoadhesion

To evaluate duration of mucoadhesion, an in-house apparatus was applied (Figure 2) (16). The apparatus had three test cells; two lower and upper platforms were placed in each of them. Each test cell was filled with phosphate buffer (pH 6.8). Sheep buccal mucosa was placed on the lower platform and the tablet was clung to the upper platform. The mucosa and tablet were then placed in contact with each other and a constant force by fingertip was applied for 1 min to them. Next, through two pulley systems, a 15.0 g weight was applied to each upper platform (this weight was chosen through initial studies). As soon as the tablet was separated from the mucosal surface, a small flap dropped onto a photocell detector, stopping the timer device (recording the elapsed time to 0.1 min) and measured the duration of mucoadhesion of the tablet. Each experiment was run in triplicate, and the results were expressed as mean ± SD.

*In-vitro drug release kinetic studies*

In order to find out the release kinetics of drug from chosen formulations, data obtained from *in-vitro* drug release experiment were fitted into different kinetic mathematical models such as zero order and first order kinetic models (17), Higuchi model (18), Hixson-Crowell model (19) and Korsmeyer-Peppas model (the power law) (20). The equations relevant to these models are stated in Table 2. The parameters in these equations were completely described in the literature (17-20).

*Statistical Analysis*

ANOVA, Tukey *post-hoc* test, and independent sample *t*-test were applied to determine statistical signiﬁcance of data. Differences were considered to be signiﬁcant for values of *p *< 0.05. SPSS Statistics software package version 21.0 was employed for data analysis. 

## Results and Discussion

*Preparation and characterization of propranolol HCl buccoadhesive tablets*

Based on preliminary studies on the propranolol hydrochloride drug powder, it was found that the drug powder had a carr’s index value of 1.24 as well as a hausner’s ratio of 1.01. This would mean that the propranolol hydrochloride powder had excellent flow. In addition, propranolol hydrochloride powder was placed without any excipient into the tablet press die and compressed. It was found that the propranolol hydrochloride powder had suitable compressibility.

All tablets were prepared by direct compaction method and physical characteristics of them were evaluated (Table 3). The thickness of tablets, depending on the type of polymer, was found to be in the range of 2.03 ± 0.05 mm to 2.41 ± 0.05 mm. The results of tablets hardness evaluation indicated that all tablets had the adequate mechanical strength for resistance to fracture during handling. Except formulation F_10_, which had the lowest hardness, all the formulations had desired friability value (less than 1%). Weight variations of different formulations were found to be satisfactory.

*Measurement of the mucoadhesive strength*

The mucoadhesive strengths of propranolol HCl buccoadhesive tablets are given in Table 4. The study has shown the polymer type and its amounts affected mucoadhesive strength. Increasing in polymer amount caused an increase in mucoadhesive strength, because of elevating active functional groups that play a key role in linking and connecting to the mucous (21).

PEG 6000 had the lowest mucoadhesive strength among the polymers (*p *< 0.05, ANOVA and Tukey *post-hoc* test). This was probably due to low molecular weight of this polymer. It has been shown that by increasing the molecular weight of water-soluble polymers to more than 100,000, the mucoadhesive strength also increases. Polymers with highly linear configuration such as polyethylene glycols with molecular weight of 20,000 do not possess adhesive properties, but when the molecular weight increases to 200,000, the mucoadhesive strength improves (22). As shown in Table 4, the mucoadhesive strength of PEO 8000000, polyethylene glycol with high molecular weight, is much more than PEG 6000 (*p *< 0.05, independent sample *t*-test). 

The highest mucoadhesive strength belonged to carbomer 934P (*p *< 0.05, ANOVA and Tukey *post-hoc *test). This polymer is a member of poly (acrylic acid) family and it can interact with mucosa via forming hydrogen bond (23). Following carbomer 934P, HPMC K4M, a neutral polymer, had the second highest mucoadhesive strength (*p* < 0.05, ANOVA and Tukey *post-hoc* test). Mucoadhesive performance of non-ionic polymers is typically weaker than polyelectrolytes (8, 24-26). The results obtained from this study are in agreement with this statement. There is no proton-donating carboxyl group in HPMC K4M. Therefore, less mucodhesive strength of it compared to carbomer 934P may be attributed to this matter (27). The use of physical mixture of HPMC K4M with carbomer 934P in one formulation was not effective in boosting mucoadhesive strength. The interaction between these polymers and complex formation probably caused a decrease in active functional groups. Based on these results, F_1_, F_2_, F_4_, F_5_, F_7_, F_11_, and F_13_ were selected for further assessment.

*Determination of in-vitro drug release profiles*

The results showed that lactose, PEO, and HPMC K4M had no UV absorbance in the wavelength range between 200-400 nm, but the UV absorbance at the wavelength of 216 nm for carbomer 934P was observed. It should be noted that there were two wavelengths of maximum for propranolol HCl including 218 and 291 nm. Hence, the wavelength of 291 nm was used in analytical studies to prevent absorption interference.

**Figure 3:**
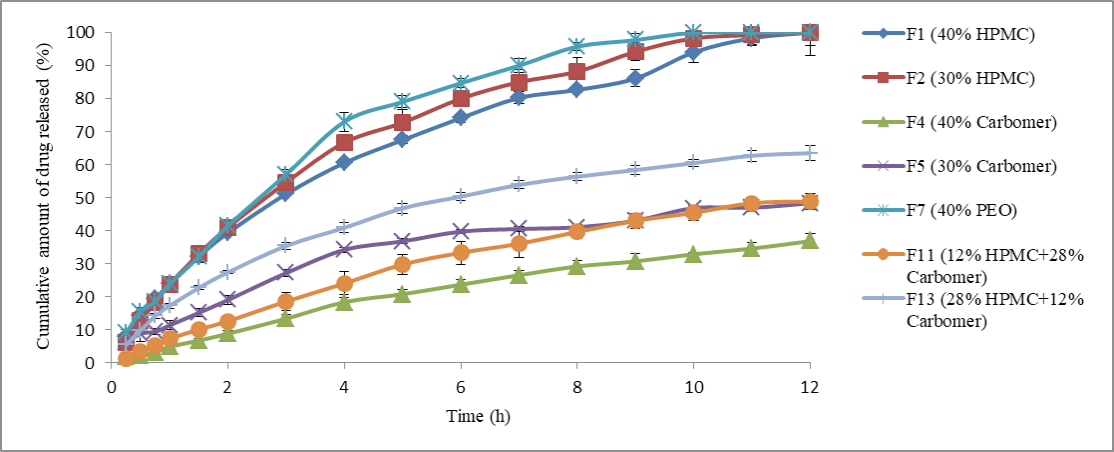
*In-vitro* release profiles of propranolol HCl buccoadhesive tablet formulations F_1_, F_2_, F_4_, F_5_, F_7_, F_11_ and F_13_ (n = 3, mean ± SD).

As previously stated, propranolol HCl buccoadhesive formulations F_1_, F_2_, F_4_, F_5_, F_7_, F_11_, and F_13_ were selected and their *in-vitro* drug release profiles were determined. The results of this test are shown in Figure 3. As can be clearly observed, formulations F_1_ and F_2_ that contained 40% of HPMC K4M and 30% of HPMC K4M respectively, released 100% of their drug content over 12 h. Formulation F_7_ that contained 40% of PEO released 100% of its drug content over 10 h, but the formulations containing carbomer 934P (four other formulations) released their drug content in a slower manner compared to the formulations containing HPMC K4M as only mucoadhesive polymer (*p* < 0.05, ANOVA and Tukey *post-hoc* test) and none of them could completely release their drug content over 12 h. With increasing the amount of HPMC K4M in combination formulation F_13_, the more amount of drug released compared to another combination formulation F_11_ (*p *< 0.05, Independent sample *t*-test). This observation may be relevant to the formation of ionic complexes between propranolol HCl (a cationic drug) and Carbomer 934P (an anionic polymer) so that the less amount of propranolol HCl molecules are available to release. The similar results were obtained in the previous study (6). In that study, complex formation between propranolol HCl and sodium alginate (an anionic polymer) reduced the release of drug molecules from vagino-adhesive propranolol HCl gel. These results could probably be in agreement with Badawi *et al.* study (28). They demonstrated that the polymers with reacting site could affect the drug release from matrix. Based on their results, by increasing the amount of anionic methacrylate copolymer, the release amount of p-amino salicylic acid from its tablets made of solid dispersions with the anionic methacrylate copolymer decreased due to complex formation between p-amino salicylic acid and polymer.

*In-vitro swelling study*

**Figure 4:**
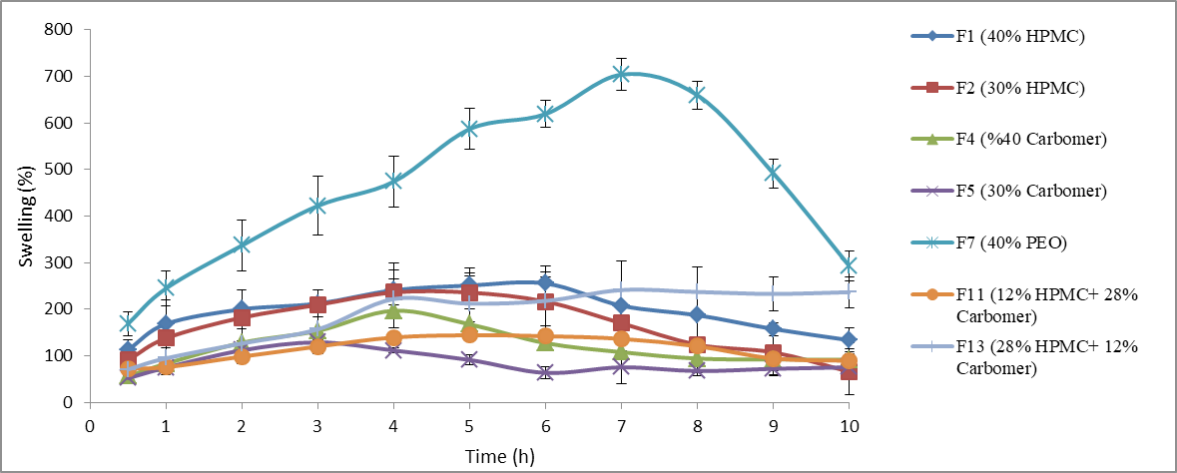
*In-vitro* swelling profiles of propranolol HCl buccoadhesive tablet formulations F_1_, F_2_, F_4_, F_5_, F_7_, F_11_ and F_13_ (n = 3, mean ± SD).

As stated in the previous section, swelling index of 7 chosen formulations was calculated. The results are shown in Figure 4. Based on the results, the formulation F_7_ containing PEO had the highest swelling index among the polymers over 9 h of the test (*p *< 0.05, ANOVA and Tukey* post-hoc* test) and the lowest swelling index nearly belonged to formulation F_5_ containing 30% of carbomer 934P. In swelling experiment, the surface layer of tablet attracts water and forms a gel layer. The characteristics of this primary gel are determinative for the continuation of swelling. Since carbomer 934P has the highest viscosity compared to HPMC K4M and PEO 8000000, the resulting gel of it is denser and for this reason the water entrance to inner layers of the tablet containing this polymer decreases. 

The swelling behavior is crucial for adhesion process (29). There is an optimal limit for hydration so that overhydration of polymer can cause adhesive joint failure (30). Overhydration of PEO led to fragmentation of the tablets and reduction in swelling index after 7 h. As mentioned, 40% of PEO had lower mucoadhesion strength than 40% of carbomer 934P or 40% of HPMC K4M. One of reasons for this observation may be attributed to overhydration of PEO.

It is well recognized that swelling and polymer erosion are prominent parameters in drug release (24). The results obtained from this test confirm the results of release test so that formulations with high swelling indexes (F_1_, F_2_, and F_7_) released all their drug content over 12 h. The amount of swelling, in addition to the amount of drug release, also affects the final size of the tablet. Buccoadhesive tablets are placed in the mouth, typically between upper lip and gum; therefore the tablets which have high swelling indexes are not suitable. Based on these results and the results obtained from drug release profiles, physicochemical properties, and mucoadhesive strength studies, two formulations F_1_ and F_2_, containing HPMC K4M, were selected for further studies.

*Determination of drug content*

Drug content of both formulations F_1_ and F_2_ was evaluated. Drug content of formulations F_1_ and F_2_ was found to be 106.8% and 93.61%, respectively. According to propranolol HCl tablet BP monograph, the tablets should not contain less than 92.5% or more than 107.5% of the active ingredient. Hence, both formulations were complied with this test.

*Determination of uniformity of dosage unit (weight variation)*

This test was performed according to pharmacopeial general chapter for uniformity of dosage units.The tablets pass this test if the calculated acceptance value relevant to the first 10 tablets is less than or equal to L_1_. Unless otherwise specified in the individual monograph, L_1_ is 15.0. Obtained acceptance values of formulations F_1_ and F_2_ were found to be 14.71 and 11.51 respectively. Hence, both formulations were confirmed by this test.

*Assessment of duration of mucoadhesion*

**Figure 5:**
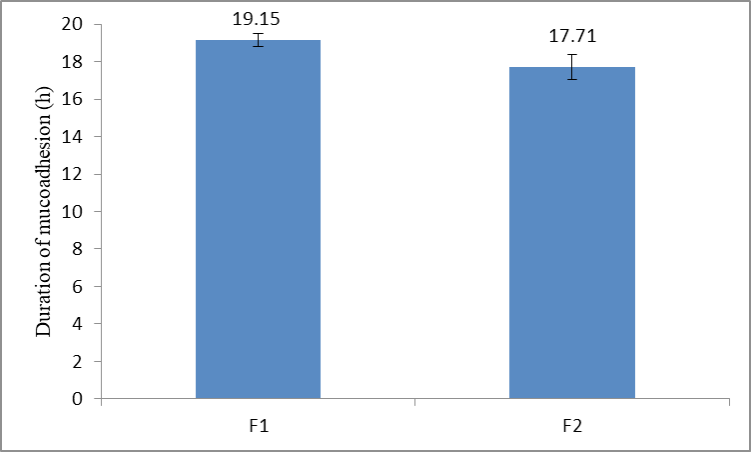
Duration of mucoadhesion of propranolol HCl buccoadhesive tablet formulations F_1_ and F_2_ (containing 40% and 30% HPMC K4M respectively) (n = 3, mean ± SD).

As was explained in a previous study, a polymer with the high mucoadhesive strength does not necessarily have a longer duration of mucoadhesion (14). Duration of mucoadhesion of two selected formulations F_1_ and F_2_ was evaluated. The results demonstrated, by applying a maximum weight of 15 g, that the buccoadhesive tablets could remain being attached to the buccal mucosa more than 17 h (Figure 5). Formulation F_1_, which had a greater amount of polymer lasted longer in contact with the mucosa compared to formulation F_2_ (*p *< 0.05, Independent sample *t*-test). Since propranolol HCl buccoadhesive tablet should remain being attached to mucosa for maximum 12 h; both formulations have been accepted in terms of duration of adhesion.

*In-vitro drug release kinetic studies*

As mentioned in the previous section, data obtained from the release experiment of formulations F_1_ and F_2_ were fitted to different mathematical models (Table 5). Data obtained from release profiles of F_1_ and F_2_ formulations fitted best into Higuchi’s model with R^2 ^values of 0.9989 and 0.9970, respectively. In order to find out the mechanism of the propranolol HCl release from buccoadhesive tablets, the first 60% drug release data were fitted in the Korsmeyer-Peppas model. The n values of formulations F_1_ and F_2_ were found to be 0.7272 and 0.8656, respectively. Based on the results, it can be concluded that the mechanism of drug release for both formulations would be based on anomalous (non-Fickian) diffusion. It means that the contribution of swelling, diffusion, and slow erosion is responsible for propranolol HCl release from buccoadhesive tablets. The erosion of tablets containing HPMC K4M was clearly observed in the swelling study. The maximum swelling for formulation F_1_ (containing 40% of HPMC K4M) and formulation F_2_ (containing 30% of HPMC K4M) was attained over 6 h and 5 h, respectively. After that the tablets began to erode slowly.

**Table 1:** Polymer and lactose composition (w/w%) in the buccoadhesive tablets of propranolol HCl

Formulation Code	HPMC K4M	Carbomer 934P	PEO 8000000	PEG 6000	Lactose	Formulation Code	HPMC K4M	Carbomer 934P	Lactose
F_1_	40	-	-	-	10	F_11_	12	28	10
F_2_	30	-	-	-	20	F_12_	20	20	10
F_3_	20	-	-	-	30	F_13_	28	12	10
F_4_	-	40	-	-	10				
F_5_	-	30	-	-	20				
F_6_	-	20	-	-	30				
F_7_	-	-	40	-	10				
F_8_	-	-	30	-	20				
F_9_	-	-	20	-	30				
F_10_	-	-	-	40	10				

**Table 2:** Different kinetic Equations used in this study

Model type	Equation
Zero order	$Q=Q_{0}+K_{O}t$
First order	logQ=logQ0-Kt2.303
Higuchi	Qt=KHt12
Hixson –Crowell	W013-Wt13=κt
Korsemeyer-Peppas model	MtM∞=Ktn

**Table 3:** Physical properties of propranolol HCl buccoadhesive tablets. Data are expressed as mean ± SD

Formulation code	Thickness (mm) n = 10	Weight variation (g) n = 10	Hardness (Kp) n = 10	Friability (%) n = 20
F_1_	2.41 ± 0.05	0.166 ± 0.004	7.13 ± 2.36	0.18
F_2_	2.16 ± 0.04	0.157 ± 0.005	6.80 ± 2.9	0.80
F_3_	2.16 ± 0.05	0.158 ± 0.004	6.43 ± 1.67	0.81
F_4_	2.17 ± 0.12	0.161 ± 0.006	14.12 ± 1.59	0.18
F_5_	2.03 ± 0.05	0.158 ± 0.004	10.93 ± 1.66	0.43
F_6_	2.06 ± 0.07	0.162 ± 0.005	9.24 ± 1.85	0.24
F_7_	2.26 ± 0.10	0.162 ± 0.004	8.70 ± 1.17	0.00
F_8_	2.23 ± 0.06	0.160 ± 0.005	7.41 ± 1.15	0.22
F_9_	2.19 ± 0.05	0163 ± 0.003	5.27 ± 1.17	0.34
F_10_	2.19 ± 0.08	0.159 ± 0.005	4.59 ± 2.33	1.18
F_11_	2.19 ± 0.14	0.163 ± 0.004	9.94 ± 1.69	0.49
F_12_	2.19 ± 0.09	0.158 ± 0.004	11.37 ± 2.89	0.16
F_13_	2.24 ± 0.09	0.159 ± 0.005	10.24 ± 0.70	0.75

**Table 4:** Mucoadhesive strength of propranolol HCl buccoadhesive tablets (n = 3, mean ± SD).

Formulation code	Mucoadhesive strength (g)	Formulation code	Mucoadhesive strength (g)
F_1_	43.93 ± 12.4	F_8_	13.08 ± 6.00
F_2_	20.00 ± 7.50	F_9_	12.87 ± 2.62
F_3_	17.67 ± 5.51	F_10_	1.59 ± 0.83
F_4_	56.67 ± 3.51	F_11_	37.67 ± 5.86
F_5_	33.00 ± 3.00	F_12_	18.67 ± 0.58
F_6_	11.00 ± 2.64	F_13_	20.33 ± 4.04
F_7_	25.48 ± 12.7		

**Table 5:** Regression values of *in-vitro* release kinetic study of propranolol HCl buccoadhesive tablets

	**R** ^2^ ** value**				
**Formulation code**	**Zero order**	**First order**	**Higuchi**	**Hixon- Crowell**	**Korsemeyer-Peppas**
F_1_	0.9431	0.9010	0.9989	0.9364	0.9972
F_2_	0.9096	0.9191	0.9970	0.9799	0.9950

## Conclusion

In conclusion, it seems that with the increase in mucoadhesive polymer amount, the mucoadhesive strength and duration of adhesion would increase. Propranolol HCl buccoadhesive formulation containing 40% of carbomer 934P possessed the highest mucoadhesion strength. However, since a good mucoadhesive system, in addition to sufficient mucoadhesion strength, should have a proper ability of drug release, it was not considered as the final chosen formulation. Formulation F1 (containing 40% of HPMC K4M) had more mucoadhesion strength than formulation F2 (containing 30% of HPMC K4M). Finally, based on the appropriate physicochemical properties, sufficient mucoadhesive strength, extended duration of mucoadhesion, adequate swelling ability, and suitable drug release profile over a period of 12 h, formulation F_1_ was introduced as the best formulation for preparing propranolol HCl mucoadhesive tablets. Although the efficiency of propranolol HCl buccoadhesive tablets, as well as mucosal irritation of them should be monitored under the *in-vivo* conditions, however, according to the results of this study, it seems that such tablets can be considered as an alternative route to bypass the first pass metabolism of propranolol HCl.

## References

[1] Raju KN, Velmurugan S, Deepika B, Vinushitha S (2011). Formulation and in-vitro evaluation of buccal tablets of Metoprolol tartrate. Int. J. Pharm. Pharm Sci.

[2] Raghavendra NG, Kulkarni GS (2012). Formulation and evaluation of mucoadhesive buccal bilayered tablets of salbutamol. Int. J. Drug. Dev. Res.

[3] Bind AK, Gnanarajan G, Kothiyal P (2013). A review: sublingual route for systemic drug delivery. Int. J. Drug. Res. Tech.

[4] Krupashree KG, Parthiban S, Vikneshwari K, Senthil kumar GP, Tamizmani T (2015). Formulation and in-vitro evaluation of mucoadhesive buccal tablets of gliclazide. Asian J. Res. Biol. Pharm. Sci.

[5] Fatima S, Panda N, Reddy AV, Fatima S (2015). Buccal mucoadhesive tablets of sumatriptan succinate for treatment of sustainable migraine: Design, formulation and in-vitro evaluation. Int. J. Pharm. Res. Allied Sci.

[6] Tasdighi E, Jafari Azar Z, Mortazavi SA (2012). Development and in-vitro evaluation of a contraceptive vagino-adhesive Propranolol hydrochloride gel. Iran. J. Pharm. Res.

[7] Aditya A, Gudas GK, Bingi M, Debnath S, Rajesham VV (2010). Design and evaluation of controlled release mucoadhesive buccal tablets of lisinopril. Int. J. Curr. Pharm. Res.

[8] Singh R, Sharma D, Garg R (2017). Review on mucoadhesive drug delivery system with special emphasis on buccal route: An important tool in designing of novel controlled drug delivery system for the effective delivery of pharmaceuticals. J. Dev. Drugs.

[9] Gandhi RB, Robinson JR (1994). Oral cavity as a site for bioadhesive drug delivery. Adv. Drug Deliv. Rev.

[10] Madgulkar A, Kadam S, Pokharkar V (2009). Development of buccal adhesive tablet with prolonged antifungal activity: Optimization and ex-vivo deposition studies. Indian J. Pharm. Sci.

[11] Patel VM, Prajapati BG, Patel HV, Patel KM (2007). Mucoadhesive bilayer tablets of propranolol hydrochloride. AAPS Pharm. Sci. Tech.

[12] Abruzzo A, Cerchiara T, Bigucci F, Gallucci MC, Luppi B (2015). Mucoadhesive buccal tablets based on chitosan/gelatin microparticles for delivery of Propranolol hydrochloride. J. Pharm. Sci.

[13] Mehravaran N, Moghimi H, Mortazavi SA (2010). The influence of various mucoadhesive polymers on in-vitro performance of the resulting artificial saliva pump spray formulations. Iran. J. Pharm. Res.

[14] Mortazavi SA (2002). A comparative study between the strength and duration of mucosaadhesion of transbuccal carbomer based aqueous gels. Iran. J. Pharm. Res.

[15] U.S.P. Pharmacopoeia-National Formulary (USP 39-NF 34) (2016). United States Pharmacopeial Convention.

[16] Mortazavi SA, Smart J (1994). An in-vitro method for assessing the duration of mucoadhesion. J. Control. Release.

[17] Costa P, Sousa Lobo JM (2001). Modeling and comparison of dissolution profiles. Eur. J. Pharm. Sci.

[18] Higuchi T (1963). Mechanism of sustained-action medication Theoretical analysis of rate of release of solid drugs dispersed in solid matrices. J. Pharm. Sci.

[19] Dash S, Murthy PN, Nath L, Chowdhury P (2010). Kinetic modeling on drug release from controlled drug delivery systems. Acta Pol. Pharm.

[20] Korsmeyer RW, Gurny R, Doelker E, Buri P, Peppas NA (1983). Mechanisms of solute release from porous hydrophilic polymers. Int. J. Pharm.

[21] Roy S, Pal K, Anis A, Pramanik K, Prabhakar B (2009). Polymers in mucoadhesive drug-delivery systems: A brief note. Des. Monomers Polym.

[22] Chen JL, Cry GN, Manly RS (1970). Compositions producing adhesion through hydration. Adhesive Biological Systems.

[23] Patel MM, Smart JD, Nevell TG, Ewen RJ, Eaton PJ, Tsibouklis J (2003). Mucin/poly(acrylic acid) interactions: a spectroscopic investigation of mucoadhesion. Biomacromolecules.

[24] Korner A, Larsson A, Andersson A, Piculell L (2010). Swelling and polymer erosion for poly(ethylene oxide) tablets of different molecular weights polydispersities. J. Pharm. Sci.

[25] Mortazavi S, Moghimi H (2010). Effect of surfactant type and concentration on the duration of mucoadhesion of carbopol 934 and hpmc solid compacts. Iran. J. Pharm. Res.

[26] Singla AK, Chawla M, Singh A (2000). Potential applications of carbomer in oral mucoadhesive controlled drug delivery system: a review. Drug Dev. Ind. Pharm.

[27] Nafee NA, Ismail FA, Boraie NA, Mortada LM (2004). Mucoadhesive delivery systems Evaluation of mucoadhesive polymers for buccal tablet formulation. Drug Dev. Ind. Pharm.

[28] Badawi AA, Fouli AM, El-Sayed AA (1980). Drug release from matrices made of polymers with reacting sites. Int. J. Pharm.

[29] Rojewska M, Olejniczak-Rabinek M, Bartkowiak A, Snela A, Prochaska K, Lulek J (2017). The wettability and swelling of selected mucoadhesive polymers in simulated saliva and vaginal fluids. Colloids Surf. B Biointerfaces.

[30] Smart JD (2005). The basics and underlying mechanisms of mucoadhesion. Adv. Drug Deliv. Rev.

